# 387. Understanding the Impact of COVID-19 on Cognitive Function Using a Scalable, App-based, Self-administered Tool in the Military

**DOI:** 10.1093/ofid/ofad500.457

**Published:** 2023-11-27

**Authors:** Liana Andronescu, Stephanie A Richard, Emily Hone, Ann Scher, David Lindholm, Katrin Mende, Anuradha Ganesan, Nikhil Huprikar, Tahaniyat Lalani, Alfred Smith, Rupal Mody, Milissa U Jones, Rhonda Colombo, Evan Ewers, Catherine Berjohn, Carlos Maldonado, Margaret Sanchez Edwards, Julia Rozman, Jennifer Rusiecki, Celia Byrne, Mark P Simons, David R Tribble, David R Tribble, Timothy Burgess, Leah H Rubin, Joan Severson, Robert O’Connell, Simon Pollett, Brian Agan

**Affiliations:** Infectious Disease Clinical Research Program, USUHS, Bethesda, Maryland; Infectious Disease Clinical Research Program, Department of Preventive Medicine and Biostatistics, Uniformed Services University of the Health Sciences, Bethesda, MD, USA, Bethesda, Maryland; Infectious Disease Clinical Research Program, USUHS, Bethesda, Maryland; Uniformed Services University of the Health Sciences, Bethesda, Maryland; Department of Medicine, Uniformed Services University of the Health Sciences; Brooke Army Medical Center, San Antonio, Texas; Brooke Army Medical Center, San Antonio, Texas; Infectious Disease Clinical Research Program, USUHS; Henry M. Jackson Foundation for the Advancement of Military Medicine Inc, Bethesda, Maryland; Walter Reed National Military Medical Center, Bethesda, Maryland; Naval Medical Center Portsmouth, Portsmouth, Virginia; Naval Medical Center, Portsmouth, Virginia; William Beaumont Army Medical Center, El Paso, Texas; Uniformed Services University, Bethesda, Maryland; Infectious Disease Clinical Research Program, USUHS, Bethesda, Maryland; Fort Belvoir Community Hospital, Fort Belvoir, Virginia; Naval Medical Center San Diego, San Diego, California; Womack Army Medical Center, Fort Bragg, North Carolina; Infectious Disease Clinical Research Program, Department of Preventive Medicine and Biostatistics, Uniformed Services University of the Health SciencesHenry M. Jackson Foundation for the Advancement of Military Medicine, Bethesda, Maryland; Infectious Disease Clinical Research Program, USUHS, Bethesda, Maryland; Uniformed Services University of the Health Sciences, Bethesda, Maryland; Uniformed Services University of the Health Sciences, Bethesda, Maryland; Infectious Disease Clinical Research Program, Department of Preventive Medicine and Biostatistics, Uniformed Services University of the Health Sciences, Bethesda, MD, USA, Bethesda, Maryland; Uniformed Services University of the Health Sciences, Bethesda, Maryland; Uniformed Services University of the Health Sciences, Bethesda, Maryland; Infectious Disease Clinical Research Program, Department of Preventive Medicine and Biostatistics, Uniformed Services University of the Health Sciences, Bethesda, MD, USA, Bethesda, Maryland; Johns Hopkins University School of Medicine, Baltimore, Maryland; Digital Artefacts, Iowa City, Iowa; Infectious Disease Clinical Research Program, USUHS, Bethesda, Maryland; Infectious Disease Clinical Research Program, Department of Preventive Medicine and Biostatistics, Uniformed Services University of the Health Sciences, Bethesda, MD, USA, Bethesda, Maryland; Infectious Disease Clinical Research Program, Department of Preventive Medicine and Biostatistics, Uniformed Services University of the Health Sciences, Bethesda, MD, USA, Bethesda, Maryland

## Abstract

**Background:**

SARS-CoV-2 infections have been associated with self-reported impaired cognitive function, but research examining objective cognitive assessments is scant. Given the potential impact of long-term cognitive impairment, it is important to characterize this post-infection phenotype.

**Methods:**

The Epidemiology, Immunology, and Clinical Characteristics of Emerging Infectious Diseases with Pandemic Potential (EPICC) study is a longitudinal cohort assessing the impact of SARS-CoV-2 infection in Military Health System (MHS) beneficiaries. A subset of EPICC enrollees consented to cognitive assessment using the Brain-Baseline Assessment of Cognition and Everyday Functioning app (BRACE; Digital Artefacts LLC, Iowa City, IA) and completed 4 tasks: Trails Making Tests A and B, Stroop task, and Visuospatial Short-term Memory task. Participants completed the tasks in August-September 2022 and were categorized as impaired if their mean completion time was >1 SD above the control sample mean.

**Results:**

A total of 482 participants completed the cognitive assessments, 71% of whom had a known history of SARS-CoV-2 infection. Among those with a history of SARS-CoV-2 infection, the mean time between first positive SARS-CoV-2 test and module completion was 9 months (SD=5). Participants were primarily active duty service members (80%), male (65%), and non-Hispanic white (70%). SARS-CoV-2 infections were primarily mild or asymptomatic with only 14 (4.1%) hospitalized. Logistic regression models adjusted for sex, race/ethnicity, age, and education showed no difference in impairment in any of the BRACE tests comparing those with and without a history of SARS-CoV-2 infection (Figure 1). Age was a risk factor for impairment across all tests with each additional year increasing risk of impairment by 6-8% (95% CI: 1.04 – 1.11).

**Figure 1.**
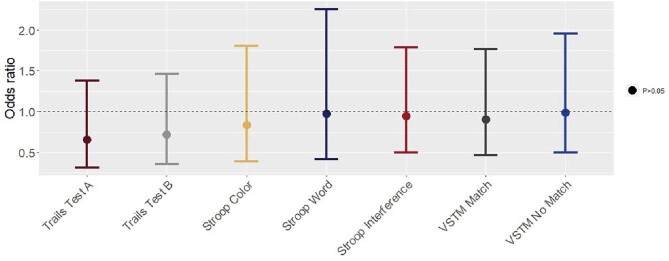
Adjusted odds of scoring >1 SD above mean completion time of SARS-CoV-2 negative participants Logistic regression adjusted for age, sex, race/ethnicity, and education

**Conclusion:**

MHS beneficiaries with a history of SARS-CoV-2 infection did not demonstrate a long-term higher prevalence of objectively measured cognitive impairment compared to participants without SARS-CoV-2 infection after adjusting for demographic variables. Further study is needed to understand the incongruence between reported cognitive symptoms and objectively measured cognitive performance.

**Disclosures:**

**Julia Rozman, BS**, AstraZeneca: TBD **Mark P. Simons, PhD**, AstraZeneca: The IDCRP and HJF were funded to conduct an unrelated phase III COVID-19 monoclonal antibody immunoprophylaxis trial as part of US Govt COVID Response **Timothy Burgess, MD, MPH**, AstraZeneca: The IDCRP and the Henry M. Jackson Foundation (HJF) were funded to conduct an unrelated phase III COVID-19 monoclonal antibody immunoprophylaxis trial **Simon Pollett, MBBS**, AstraZeneca: The IDCRP and the Henry M. Jackson Foundation (HJF) were funded to conduct an unrelated phase III COVID-19 monoclonal antibody immunoprophylaxis trial

